# H-RACS: a handy tool to rank anti-cancer synergistic drugs

**DOI:** 10.18632/aging.103925

**Published:** 2020-11-10

**Authors:** Xinmiao Yan, Yiyan Yang, Zikun Chen, Zuojing Yin, Zeliang Deng, Tianyi Qiu, Kailin Tang, Zhiwei Cao

**Affiliations:** 1 Department of Gastroenterology, Shanghai Tenth People’s Hospital, School of Life Sciences and Technology, Tongji University, Shanghai 200092, China; 2 Shanghai Public Health Clinical Center, Fudan University, Shanghai 200032, China

**Keywords:** anti-cancer, synergistic combination, drug synergy, web server, bioinformatics

## Abstract

Though promising, identifying synergistic combinations from a large pool of candidate drugs remains challenging for cancer treatment. Due to unclear mechanism and limited confirmed cases, only a few computational algorithms are able to predict drug synergy. Yet they normally require the drug-cell treatment results as an essential input, thus exclude the possibility to pre-screen those unexplored drugs without cell treatment profiling. Based on the largest dataset of 33,574 combinational scenarios, we proposed a handy webserver, H-RACS, to overcome the above problems. Being loaded with chemical structures and target information, H-RACS can recommend potential synergistic pairs between candidate drugs on 928 cell lines of 24 prevalent cancer types. A high model performance was achieved with AUC of 0.89 on independent combinational scenarios. On the second independent validation of DREAM dataset, H-RACS obtained precision of 67% among its top 5% ranking list. When being tested on new combinations and new cell lines, H-RACS showed strong extendibility with AUC of 0.84 and 0.81 respectively. As the first online server freely accessible at http://www.badd-cao.net/h-racs, H-RACS may promote the pre-screening of synergistic combinations for new chemical drugs on unexplored cancers.

## INTRODUCTION

Despite the significant efficacy, cancer monotherapy has frequently been reported with acquired drug resistance due to tumor heterogeneity [1]. In recent years, combinational therapies of drug synergy were actively sought with increased efficacy, reduced side effects, and delayed drug resistance [2–4]. Representative examples include a combination of panobinostat and doxorubicin for acute myeloid leukemia [5], and the use of histone-deacetylase inhibitor AR-42 in combination with cisplatin in bladder cancer treatment [6]. While promising, identifying synergistic drugs from a large pool of candidates remains challenging for specific cancer types. Complicated context factors have been found to affect the synergistic effects of drug treatment, such as drug structures, tested cell lines/animals, drug dosage, sequential treatment, and testing conditions, and so on [7]. Under such circumstances, searching synergistic partners via ‘trial-and-error’ experiments seem impractical considering the huge space of potential drugs with various dose combinations on different testing cell lines [8]. More cost-effective computational methods have been explored to reduce the searching landscapes of subsequent experiments.

Currently, only a few algorithms have been published with promising performance [9, 10], such as Drug-Induced Genomic Residual Effect (DIGRE) [11], IUPUI_CCBB methods [3], Combination Drug Assembler (CDA) [12], Huang’s and Parkkinen’s method [13]. In 2015, our group constructed a workflow of RACS with top performance among peers [14, 15]. Yet most models require key input of drug-cell interaction profiling, such as the pairwise change before and after drug treatment on the same cancer cell lines [16]. While the expression profiles of drug tests on cancer cell lines are still insufficient and scattered, only a limited number of drugs are viable to these models. In the pressing need for those unexplored drug combinations on unexplored cancers, more practical methods without input requirement of drug-cell-treatment have been actively desired.

According to literature searching, three such models have been reported so far. The general idea of them is to collect known synergistic (positive) and non-synergistic (negative) combinations on different cancers, then construct various features that help to differentiate positive from negative combinations. Finally, those important features are used to further build a prediction model. Specifically, the first model was Zhao’s method proposed in 2011 based on drugs information of MeSH terms, therapeutic and side effects, together with network features of the drug targets [17]. The second was from Li et al, which improved Zhao’s method in specificity and sensitivity through integrating drug similarity calculated based on drug targets [18]. It is noted that both above ignored the difference of cancer context. Soon a more comprehensive one, DeepSynergy, was reported in 2018 considering both drug and cancer information [19]. On top of the drug-related features, DeepSynergy utilized the basal expression profiles of tested cell lines with no drug treatment, which was very insightful and promising to screen new drug combinations on a wide range of cell lines. But, regrettably, the performance was not validated on any independent dataset, and low predictive performance was found on new or unexplored drug combinations or cell lines according to authors’ claim [19]. Furthermore, the page of DeepSynergy only displayed the calculated results between 38 testing drugs on 39 cell lines in the paper, providing no uploading access of interested drug list from users. In this sense, it was viewed as a “data portal” rather than a prediction tool [20]. To summarize, these published models seem still inadequate to a large-scale exploration of synergistic drugs for cancers.

In addition to important features related to drug synergy, another challenge of model performance is the data insufficiency of synergistic drugs on cancers. Till now, there are mainly three sets of experimental data on synergistic effects of anti-cancer drugs. The first was released by the Dialogue for Reverse Engineering Assessments and Methods (DREAM) consortium in 2014, regarding 91 drug combinations derived from 14 compounds on the human diffuse large B-cell lymphoma cell line OCI-LY3 [3]. The second was from O’Neil describing synergistic effects of 22,737 combinational scenarios, between 38 drugs on 39 cancer cell lines in 2016 [4]. A combinational scenario is tentatively defined as one unique drug combination on a unique cell line, regardless of dose variation. Most recently, the third data source was published by AstraZeneca in 2019 testing 995 drug combinations on 137 cell lines, producing synergistic results of 20,482 combinational scenarios [21]. It is noticed that different datasets used different standards to judge synergistic effects. The early DREAM project took excess over bliss (EOB) model by a single-dose response curve, while the latter two large datasets, AstraZeneca and O’Neil project, both judge by synergy score calculated on multiple doses response surface through Combenefit [22–25]. Hence the data of AstraZeneca and O’Neil can be integrated into a more comprehensive and representative dataset (A&O) for further model construction and testing.

In this work, we built a handy tool, H-RACS, to predict synergistic drug combinations on cancers based on the largest data of the A&O dataset. External validations were made on different sets of independent data, including A&O, DREAM data, and those unexplored drug combinations and cell lines respectively. Finally, a web server was made publicly available as the first online tool to predict drug synergy for community applications in cancer area.

## RESULTS

### Model construction and validation

**Figure 1:**
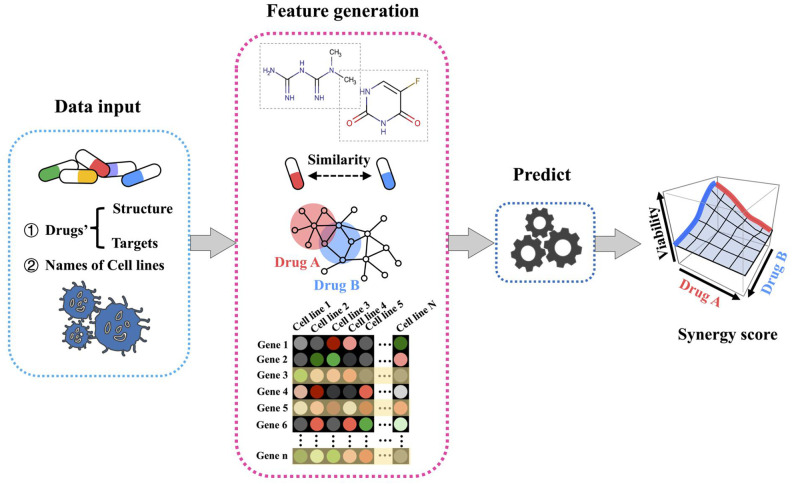
**Workflow for H-RACS illustrating the steps to predict synergy score.**

H-RACS was developed to predict the synergistic potential of drug combinations on given human cancer cell lines, the modelling workflow is illustrated in Figure 1. Input datasets include drug structures, corresponding target lists, and names of cell lines. The features of H-RACS are composed of drug chemical descriptors, drug similarities, drug targeting network features, and signature genes of basal cell lines. The synergy score is calculated as the final output.

Seven machine learning models were firstly trained based on two-thirds of the A&O dataset, including 22,382 combinational scenarios. The remaining 11,192 combinational scenarios were adopted for the independent test. Internal five-fold cross-validation was executed for each model. The model performance was evaluated qualitatively by classifying synergistic or non-synergistic scenarios, and quantitatively by synergy score regression. For classification, the overall model performance of area under the receiver operator characteristics curve (AUC) and accuracy (ACC) were adopted as parameters for evaluation. For regression, the Root Mean Squared Error (RMSE) and consistency of R Squared (R^2^) were adopted between predicted and experimental results. The higher of AUC, ACC and R^2^, the better performance of a model. And for RMSE, the lower the better.

**Figure 2:**
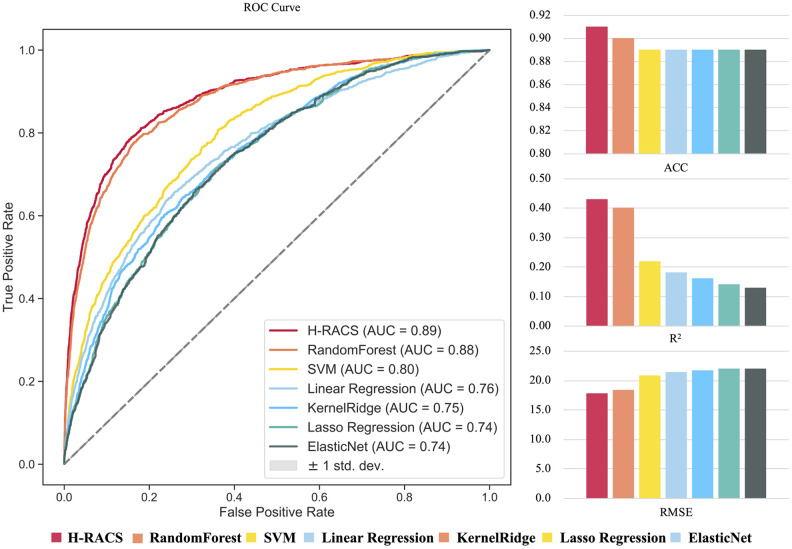
**The performance comparison of seven models based on the independent validation dataset.** Model performance is evaluated by AUC, ACC, R^2^ and RMSE respectively.

Among the seven models, Gradient Boosting Regression gave the highest AUC (0.87), ACC (0.91), R^2^ (0.40), and the lowest RMSE (18.43), hence was chosen for H-RACS (Supplementary Figure 1). More details of testing models can be found in Supplementary Table 1. As the first independent validation, H-RACS was tested on the remaining one-third of A&O combinational scenarios. The AUC of 0.89 and ACC of 0.91 in classification were obtained on the remaining 11,192 scenarios (Figure 2), exhibiting high predictive performance. Further, among the seven models, the overall RMSE between predicted and experimental synergy score is the lowest of 17.78 in regression, and R^2^ is the highest, showing the best consistency between them.

### High precision on DREAM challenge data

**Figure 3:**
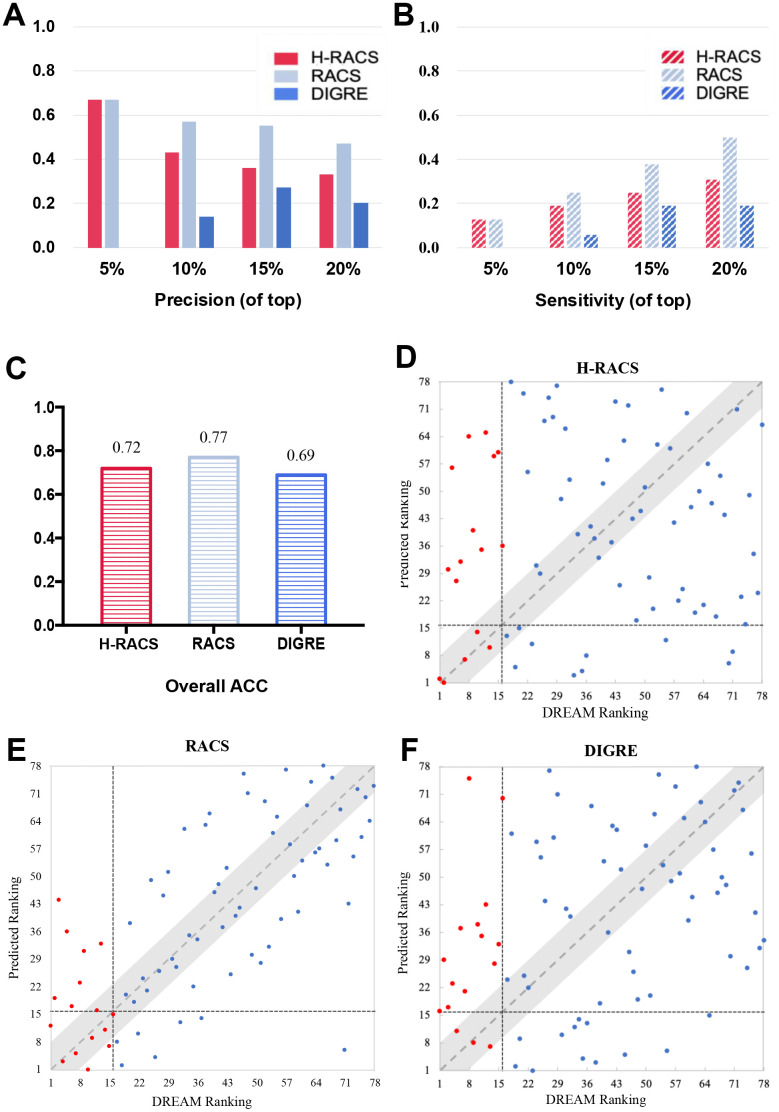
**Models’ comparison on the DREAM challenge dataset.** (**A**) The precision at top 5%, 10%, 15%, 20% ranked combinational scenarios of H-RACS, RACS and DIGRE; (**B**) The sensitivity at top 5%, 10%, 15%, 20% ranked combinational scenarios, and overall accuracy of H-RACS, RACS and DIGRE; (**C**) The overall accuracy of H-RACS, RACS and DIGRE; (**D**–**F**) The detailed ranking agreement between the predicted results and DREAM experimental results, The red dots are true synergistic drug combinations, while the blue dots are the non-synergistic ones confirmed from DREAM experiments. The vertical black dashed lines indicate the boundary between the top 16 synergistic pairs and non-synergistic ones, while the horizontal black dashed line illustrates the boundary between the top 16 predicted ranking and the rest 62 ones.

Furthermore, H-RACS was tested on a new set of data from the DREAM challenge [3]. Here, 78 drug combinations derived from 13 compounds were predicted by H-RACS because Mitomycin C is a potent DNA crosslinker and its targets have not been specified (Supplementary Table 2) [26]. The model’s ability to score positive combinational scenarios into the top-ranking list was evaluated by the accuracy, precision, and sensitivity at different cut-offs. It can be seen from Figure 3A that, H-RACS obtained a high precision of 0.67, 0.43 and 0.33 at different cutoffs of 5%, 10% and 20% of the top ranking lists respectively. Most importantly, the two most synergistic pairs in experiments were scored exactly into the top two by H-RACS (Figure 3D).

The performance of H-RACS was compared with that of the best model DIGRE from the DREAM challenge and the current best model RACS on the DREAM dataset. The performances of DIGRE and RACS were retrieved from previous literature and are shown in Figure 3 [15]. It is worth noting that both of them require essential input of the expression profiling change after the drug treatment. Despite the different loading of input requirement, H-RACS outperformed DIGRE on precision and sensitivity at different cut-offs of the top-ranking list, as well as the overall ACC.

In comparison with RACS, RACS still performed better than H-RACS on most parameters. Yet H-RACS performed as good as RACS with the precision of 0.67 for the top 5% ranking list. Interestingly, only H-RACS captured the two most synergistic combinations in the DREAM challenge, which was missed out by both RACS and DIGRE (Figure 3E, 3F), indicating the outstanding potential of H-RACS to identify the most synergistic drug combinations even with less load of input data.

### Outstanding performance on unexplored drug combinations or cell lines

To test the predictive performance of H-RACS on unexplored drug combinations, we randomly split A&O data into about 2/3 for training and 1/3 for external testing datasets by the function of GroupShuffleSplit in the module of sklearn.model_selection, considering both the number of combinational scenarios and non-redundant drug combinations [27]. Among the A&O dataset of 33,574 combinational scenarios covering 1,380 drug combinations and 116 cell lines, 920 unique drug combinations (22,320 scenarios) were set as training data, while the remaining 460 drug combinations (11, 254 scenarios) distinct from training combinations were kept as the external unexplored drug combinations to test H-RACS independently (Supplementary Table 3).

Similar data splitting was performed for unexplored cell lines. Among the A&O dataset, 77 unique cell lines (22,680 scenarios) were set as training data, while the remaining from 39 cell lines (10,894 scenarios) non-overlapping with training cell lines were kept as independent testing of unexplored cell lines i lines (Supplementary Table 3). The testing results are shown in Table 1. H-RACS achieved high classification performance with AUC of 0.84 and ACC of 0.90 on independent data of unexplored drug combinations. Additionally, on external unexplored cell lines, H-RACS still gained AUC of 0.81 and ACC of 0.89, suggesting its ability to recommend synergistic combinations for those unexplored drugs or unexplored cell lines.

**Table 1:** Predictive performance of H-RACS on unexplored drug combinations and cell lines.

**Test on**	**Validation**	**Classification**		**Regression**	
		**AUC ^a^**	**ACC ^b^**	**RMSE ^c^**	**R^2^^d^**
Unexplored drug combinations	Internal	0.87±0.01	0.90±0.00	18.00±0.50	0.40±0.02
	External	0.84	0.90	19.64	0.36
Unexplored cell lines	Internal	0.88±0.01	0.91±0.00	18.21±0.30	0.44±0.01
	External	0.81	0.89	21.43	0.21

^a^ Area Under ROC curve

^b^ Accuracy

^c^ Root Mean Squared Error

^d^ R Squared

## DISCUSSION

Predicting drug combinations with synergistic anti-cancer effects has long been desired but remains highly challenging. One reason lies in the inherent complexity of drug synergy, where synergistic effects occur in a highly context-dependent manner. The mechanism was only roughly suggested as pharmacodynamics or pharmacokinetic related, while more details deserve further investigation [28]. Meanwhile, the criteria to judge drug synergy is still under development. According to the literature, drug synergy could be judged by CI index, or synergy score, or others [23, 29]. Even the CI index can be derived from different models such as Bliss independence model [3, 30], Loewe additivity [24], highest single agent (HSA) [31], median-effect [32, 33], which further complicates the data cleaning when selecting benchmark and testing datasets. On the same combinational scenarios, this often leads to a contrast conclusion of synergy or not. In this work, we just took the well-standardized high-throughput data as training and testing datasets to avoid the inconsistency.

From recent testing, drug related features, cell line related features, and drug-cell-interaction features seem to all contribute to synergy prediction [9–15]. Those top ranking models require drug-cell-interaction profiling as a key input, while the profiling/-omics change is publicly available for only a small number of drugs on limited cell lines. Considering the enormous chemical space of interested candidates with treatment profiling yet to be explored, pre-screening algorithm is in urgent need as an initial hint for further experiments. Here we proposed a handy tool, H-RACS, to achieve the above goal. Taking gene signatures of basal cell lines without drug perturbation, instead of the profiling change before and after drug-cell-treatment, H-RACS achieved an impressive performance on different sets of independent testing data. In particular, on the DREAM challenge dataset, it outperformed model DIGRE (the best in DREAM challenge) [3], being slightly inferior to RACS, currently the best on this dataset [15]. The excellent performance of H-RACS may benefit from the A&O dataset. In total, A&O dataset of 33,574 combinational scenarios covered 135 drugs and 116 cell lines for 24 cancers. Each scenario was tested multiple times in order to define the extent of synergistic effects. The data quality, standardized format, diversity and abundance provided a solid benchmark to set up machine learning model for further extension.

Despite the significant correlation with drug synergy [9], the drug-cell-treatment profiling was purposely avoided here to increase the model extendibility and portability for unexplored drugs or cancers. From the validation on DREAM challenge data, it can be seen that H-RACS paid a slight price of performance drop (ACC 0.05) as a compromise. Now with build-in profiling of 928 cell lines covering 24 common cancers, H-ARCS only needs users to upload drug information before initiating large-scale pre-screening between any interested drugs on selected cancer cell lines. For more refined prediction, we suggest user use the full version of RACS when drug-cell-treatment profiling is accessible [15].

It is aware that the current model is only applied to chemical drugs. With the subsequent updating of drug synergy data and development of common standards to define synergistic effects, the model is expected to be improved by introducing antibody drugs. Also, further efforts will focus on incorporating additional parameters to enhance the performance, such as the x-omics profiling of cancers, drug adverse effects, drug dosage and others.

In summary, we proposed a handy tool, H-RACS, to predict drug synergy for cancers. It enables pre-screening between unexplored drugs on 928 cell lines covering 24 cancers for general users in cancer community. The advantages of H-RACS lie in low requirement of data input, outstanding prediction, sensitive context of cancer subtypes, and most importantly, the extendibility to unexplored drugs or cell lines. Though further tests are still needed before going to clinic applications, the high-throughput recommendation system of H-RACS may help to reduce experimental cost and increase the searching efficiency, so as to facilitate the identification of synergistic anti-cancer therapies.

## MATERIALS AND METHODS

### Datasets and integrating

Three major datasets were involved in this study: the AstraZeneca dataset, the O’Neil dataset, and the DREAM challenge dataset. The authorized dataset from AstraZeneca was downloaded from AstraZeneca-Sanger Drug Combination Prediction DREAM Challenge. And the O’Neil dataset was downloaded from DeepSynergy [19]. The DREAM challenge dataset released in 2014 was downloaded from the supplementary of RACS [15]. After the quality check, 10,837 combinational scenarios from AstraZeneca were integrated with the O’Neil dataset into A&O data covering 33,574 combinational scenarios comprising 1,380 drug combinations and 116 cell lines (Supplementary Table 4, Supplementary Figure 2A–2D). According to the previous publication, those with a synergy score above 30 were collected as positive scenarios, and the left were negative ones [19]. The A&O dataset is used for model construction and validation. The Dream dataset is used for further independent validation.

Drug SMILES files were downloaded from DrugBank [34] and PubChem [25]. Drug targets were collected from DrugBank [34], PubChem [25], Therapeutic target database (TTD) [35] and DGIdb [36]. Drug targeting network was retrieved based on the background protein-protein interaction (PPI) network integrating six online PPI databases (HPRD [37], MINT [38], IntAct [39], BioGRID [40], DIP [41], MIPS [42]) [37–42]. The raw expression dataset of cancer cell lines was obtained from the Cancer Cell Line Encyclopedia (CCLE) project in GEO database (accession number: GSE36133) [33].

### Features construction and selection

For each combinational scenario, features related to drugs and cancer cell lines were calculated respectively for modelling. Drug features are composed of chemical descriptors, compounds similarities and network characteristics of drug targets. A total of 196 chemical descriptors were calculated based on the chemical structures by RDKIT [43]. Compound’s similarities were described via topological fingerprint similarity, Atom Pairs similarity, and Morgan Fingerprints similarity by RDKIT [43]. Drug targeting network was constructed and seven features of targeting network were calculated between drug combinations as they were previously reported for drug combination prediction [15]. For cancer cell lines, the raw expression profiles of cancer cell lines were preprocessed and quantile normalized [44]. FARMS method was used to call informative genes as the signature genes [45, 46]. 3,988 signature genes were derived from the 116 cell lines covering 11 different cancer types.

Thus, the initial feature set of 4,390 vectors includes 406 drug-related features and 3,984 signature genes of cell lines. Then feature selection was performed by two steps. Firstly, those blank features in 90% combinational scenarios were removed. Secondly, those features correlated with synergy scores were ranked and selected as the final feature set. Top 10% 20% 30% and 50% top ranked features were tested and the top 30% vectors were chosen considering both the performance and efficiency. The final feature set of 1,275 vectors covers 208 drug-related features and 1,067 signature genes of cell lines.

### Methods

Seven popular machine learning models were screened, including gradient boosting regression, random forest, support vector machine, linear regression, elastic net, kernel ridge regression, and lasso regression. The implementation of all methods is based on scikit-learn [27].

### Performance metrics

The model performance was evaluated by metrics of regression and classification respectively, including Root Mean Squared Error (RMSE), R Squared (R^2^), and Receiver Operating Characteristic (ROC) curve, the area under the receiver operator characteristics curve (AUC), accuracy (ACC) typical for classification evaluation.

### Root Mean Squared Error (RMSE)

This parameter provides a measure of the standard deviation of prediction errors:

$$RMSE\left(y_{true},\;y_{pred}\right)=\;\sqrt{\frac{1}{\;N\;}\overset{n}{\underset{i=1}{\sum }}{\left(y_{true\_i}-y_{pred\_i}\right)}^{2}}$$ (1)

Where *y_pred_i_* is the synergy score predicted for the i-th combinational scenario, and *y_true_i_* is the corresponding synergy score experimentally validated. N and n is the number of combinational scenarios for prediction.

### R squared (R^2^)

This parameter provides a measure of the correlation between predictions and experimentally validated synergy scores:

$$R^{2}\left(y_{true},\;y_{pred}\right)\;=1-\frac{\sum_{i=1}^{n}{\left(y_{true\_i}-y_{pred\_i}\right)}^{2}}{\sum_{i=1}^{n}{\left(y_{true\_i}-\bar{y}\right)}^{2}}$$ (2)

$$Where\;\;\bar{y}\;=\frac{1}{\;N\;}\overset{n}{\underset{i=1}{\sum }}y_{true\_i}$$ (3)

Where *y_pred_i_* is the synergy score predicted for the i-th combinational scenario, and *y_true_i_* is the corresponding synergy score experimentally validated. $\bar{y}$ is the mean experimentally validated synergy scores of all combinational scenarios, N and n is the number of combinational scenarios for prediction.

### Classification evaluation

Receiver Operating Characteristic (ROC) curve, the area under the receiver operator characteristics curve (AUC) and accuracy (ACC) were plotted and calculated to evaluate the model’s performance in classification [27].

## Supplementary Material

Supplementary Table 1

Supplementary Table 3

Supplementary Table 4
