# Supplementary material for: H-RACS: a handy tool to rank anti-cancer synergistic drugs
Source: Aging (Albany NY). 2020 Nov 10;12(21):21504–17. doi: 10.18632/aging.103925 (PMC7695372; doi:10.18632/aging.103925)
Supplement: Supplementary Table 1 [file aging-12-103925-s001..pdf]

Supplementary Table 3. Data description for testing H-RACS on unexplored drug combinations and cell lines.

Category	Data usage	Combinational scenarios	Drug combinations	Cell lines
A&O dataset		33,574	1,380	116
unexplored drug combinations	Training	22,320	920	116
	External test	11,254	460	116
unexplored cell lines	Training	22,680	1380	77
	External test	10,894	1378	39
