# Supplementary material for: H-RACS: a handy tool to rank anti-cancer synergistic drugs
Source: Aging (Albany NY). 2020 Nov 10;12(21):21504–17. doi: 10.18632/aging.103925 (PMC7695372; doi:10.18632/aging.103925)
Supplement: Supplementary Table 3 [file aging-12-103925-s002..pdf]

SUPPLEMENTARY FIGURES

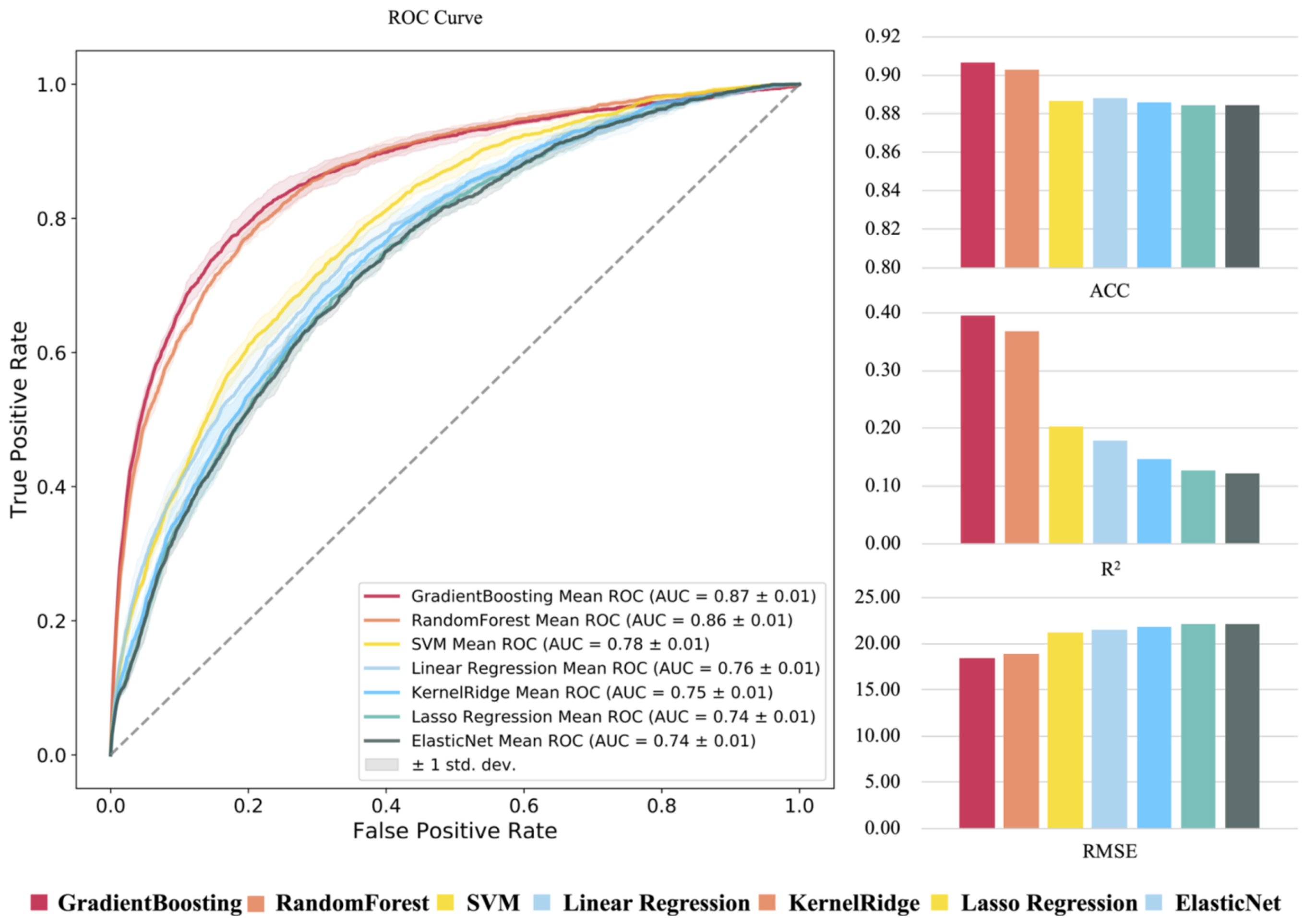

Supplementary Figure 1. The performance comparison of seven models based on five-fold cross-validation on the internal training dataset.

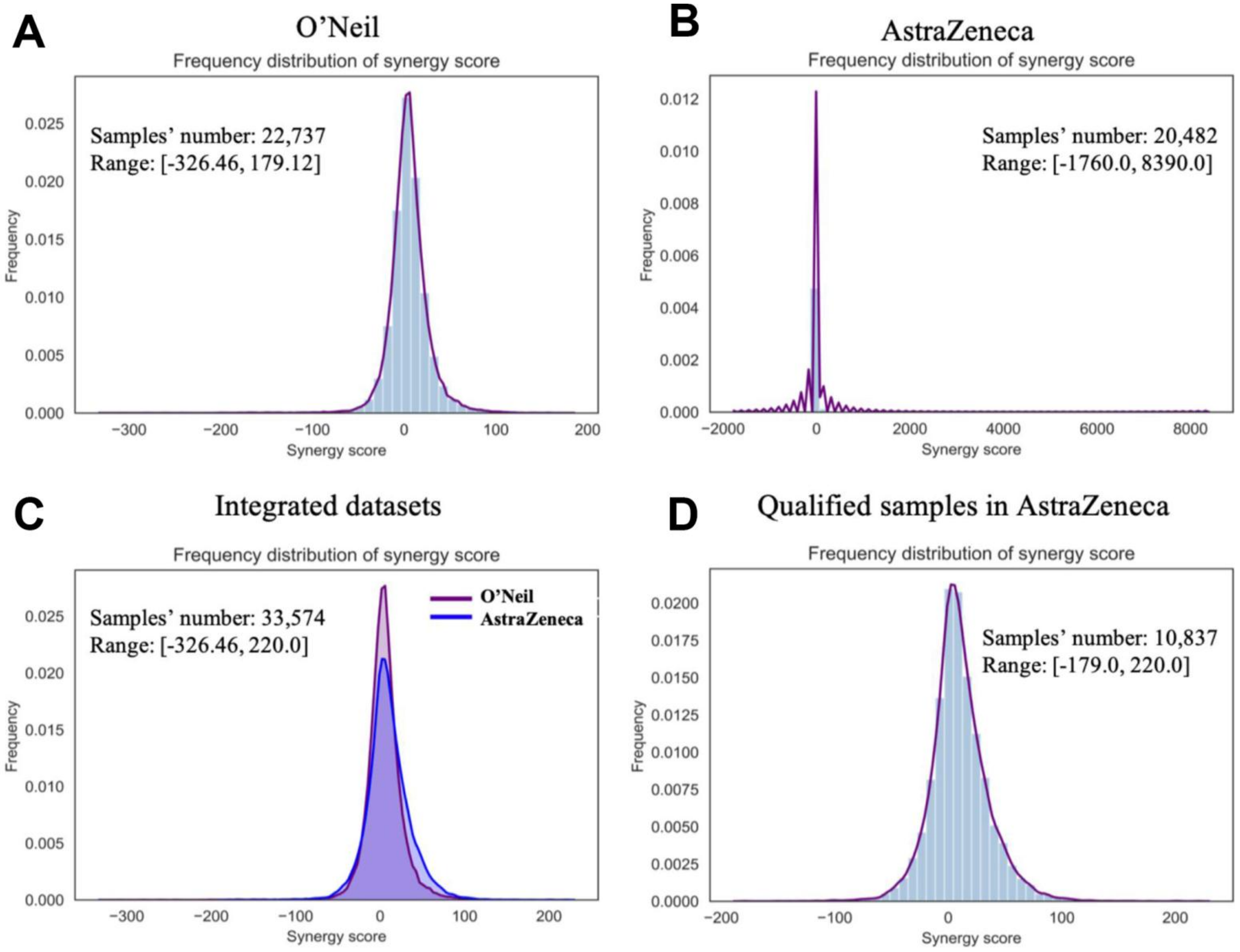

Supplementary Figure 2. Frequency distributions of synergy scores of A&O datasets. (**A**) Frequency distribution of synergy score of O'Neil dataset; (**B**) Frequency distribution of synergy score of AstraZeneca dataset; (**C**) Frequency distribution of synergy score of A&O dataset; (**D**) Frequency distribution of synergy score of qualified samples in AstraZeneca dataset.
